# A tyrosine phosphorylation switch controls the interaction between the transmembrane modulator protein Wzd and the tyrosine kinase Wze of *Lactobacillus rhamnosus*

**DOI:** 10.1186/s12866-015-0371-2

**Published:** 2015-02-21

**Authors:** Hye-Ji Kang, Christophe Gilbert, Frédérique Badeaux, Danièle Atlan, Gisèle LaPointe

**Affiliations:** STELA Dairy Research Centre, INAF, Université Laval, Québec, G1V 0A6 QC Canada; CIRI-U1111 INSERM- UMR5308 CNRS-UCBL-ENSL, Université de Lyon, Université Lyon 1, 10 rue Dubois, bât. Lwoff, F-69622 Villeurbanne cedex, France; CNRS, UMR5240, Unité microbiologie, adaptation et pathogénie, Villeurbanne, F-69622 France; Present address: Academy of Immunology and Microbiology (AIM), Institute for Basic Science (IBS), Pohang, 790-784 Republic of Korea

**Keywords:** Exopolysaccharide, Tyrosine phosphorylation, Co-polymerase, Transmembrane Modulator, Kinase, *Lactobacillus rhamnosus*

## Abstract

**Background:**

One proposed mechanism for assembly of secreted heteropolysaccharides by many Gram positive bacteria relies on the coordinated action of a polymerization complex through reversible phosphorylation events. The role of the tyrosine protein kinase transmembrane modulator is, however, not well understood.

**Results:**

The protein sequences deduced from the *wzb*, *wzd* and *wze* genes from *Lactobacillus rhamnosus* ATCC 9595 and RW-9595 M contain motifs also found in corresponding proteins CpsB, CpsC and CpsD from *Streptococcus pneumoniae* D39 (serotype 2). Use of an anti-phosphotyrosine antibody demonstrated that both Wzd and Wze can be found in tyrosine phosphorylated form. When tyrosine 266 was mutated to phenylalanine, WzdY266F showed slightly less phosphorylated protein than those produced by using eight other tyrosine mutated Wzd genes, when expressed along with Wze and Wzb in *Lactococcus lactis* subsp. *cremoris* MG1363. In order to demonstrate the importance of ATP for the interactions among these proteins, native and fusion Wzb, Wzd and Wze proteins were expressed and purified from *Escherichia coli* cultures. The modulator protein, Wzd, binds with the phosphotyrosine kinase Wze, irrespective of its phosphorylation status. However, Wze attained a higher phosphorylation level after interacting with phosphorylated Wzd in the presence of 10 mM ATP. This highly phosphorylated Wze did not remain in close association with phosphorylated Wzd.

**Conclusion:**

The Wze tyrosine kinase protein of *Lactobacillus rhamnosus* thus carries out tyrosine phosphorylation of Wzd in addition to auto- and trans- phosphorylation of the kinase itself.

**Electronic supplementary material:**

The online version of this article (doi:10.1186/s12866-015-0371-2) contains supplementary material, which is available to authorized users.

## Background

Bacteria-host interactions are modulated by cell surface structures such as capsules and other extracellular polysaccharides (EPS). These polymers are generally considered to have a protective role against adverse environmental conditions [1]. Surface polysaccharides also play a role in recognition, and thus contribute to evading the host immune system by both pathogenic and commensal bacteria. The EPS from *Lactobacillus rhamnosus* RW-9595 M stimulates interleukin, tumor necrosis factor and interferon gamma in mouse splenocytes [2], thus demonstrating a role in immunomodulation. Many strains of *L. rhamnosus* produce exopolysaccharides, but production levels vary greatly among strains. Strain RW-9595 M has shown the highest level of EPS production for a lactic acid bacterial species at 2000 mg/L under controlled pH conditions while strain ATCC 9595 produces a low level of EPS (116 mg/L) [3].

Polysaccharides secreted by Gram positive bacteria are synthesized by either a processive or a non-processive mechanism [4]. The Wzy-dependent non-processive mechanism requires the coordinated action of intracellular and membrane proteins with extracellular domains [4,5]. Inside the cell, glycosyltransferases use sugar nucleotides as substrates in order to assemble individual repeating units linked to a lipid carrier such as undecaprenyl-phosphate. When complete, each repeating unit is then transferred to the outer surface of the membrane, where the repeating units are assembled by a Wzy-dependent polymerization complex [4]. These polymers can then be covalently linked to the cell surface as capsules (CPS) in streptococci and staphylococci or released as exopolysaccharides (EPS) by lactic acid bacteria such as lactococci, streptococci or lactobacilli [5].

As components of the Wzy-dependent mechanism, the proteins proposed to be involved in determining the chain length of surface polysaccharides include a protein tyrosine phosphatase (PTP) and a polysaccharide co-polymerase (PCP) consisting of two domains, a cytosolic protein tyrosine kinase domain (PTK) and a tyrosine-protein kinase transmembrane activator or modulator (TKM) [4,5]. Polysaccharide co-polymerases have been classified into four subfamilies according to their sequence features [6]. The two domains are found in a single protein in Gram negative bacteria (subfamilies PCP1, PCP2a and PCP3), while they are coded by two separate proteins in Gram positive bacteria (PCP2b subfamily). In *Streptococcus pneumoniae,* three proteins with homologous functions named CpsB (PTP), CpsC (TKM) and CpsD (PTK) have been proposed to have roles in CPS polymerization and attachment [6]. Functional studies in *S. pneumoniae* have shown that tyrosine phosphorylation and dephosphorylation of CpsD have an impact on CPS production [7-9]. A stable complex consisting of CpsB, CpsC, CpsD and ATP is proposed to enhance capsule synthesis in *S. pneumoniae* strain D39 [8]. The level of phosphorylated CpsD (PTK) is positively correlated with capsule amount in strain D39 [7] and attachment of CPS to the cell wall is proposed to be enhanced by phosphorylated CpsD [6,10]. In this model, the transphosphorylation of the CpsD (PTK) requires the presence of the CpsC (TKM) and dephosphorylation of CpsD is carried out by the phosphatase CpsB (PTP), which can also inhibit the phosphorylation of CpsD. In addition to the role of facilitating CpsD phosphorylation, Morona et al. [6] have proposed that CpsC is also involved in attachment of CPS to the cell wall. Similar systems have been proposed for EPS elongation by *Lactococcus lactis* [11] and *S. thermophilus* [12]. Recent data suggests that the TKM/PTK complex plays a role in coordinating with cell wall growth by spatial regulation of capsule synthesis at the division septum in *S. pneumoniae* [13]. Furthermore, analogous chain length regulator protein Wzz is proposed to aid the Wzy polymerase by maintaining the nascent polysaccharide chain in a conformation conducive to continuing polymerization for O-antigen assembly [14].

The function of the complete polymerization complex and the precise role of each protein are not yet clearly understood, and may in fact differ among strains and species. When comparing the amino acid sequences between strains RW-9595 M and ATCC 9595, the Wzd tyrosine-protein kinase transmembrane modulator (TKM) and Wzb phosphatase (PTP) are 100% identical, whereas the Wze kinase (PTK) of the two strains differ by one amino acid (T72 in ATCC 9595 versus K72 in RW-9595 M) [3]. Our previous work has shown the phosphatase activity of Wzb from *L. rhamnosus* [15]. In the present study, the purification of the three proteins (Wzb, Wzd, Wze) is described and their interactions are demonstrated *in vitro*. The impact of each tyrosine on the phosphorylation state of Wzd is revealed *in vivo* using point mutations and expressing these proteins in *Lactococcus lactis* subsp. *cremoris* MG1363. This work shows tyrosine phosphorylation of the Wzd modulator protein from *L. rhamnosus* and the impact of this phosphorylation on its interaction with the tyrosine protein kinase Wze.

## Methods

### Bacterial strains and growth condition

Bacterial strains used in this study are listed in Table 1*. L. rhamnosus* strains were grown without agitation at 37°C in MRS medium (Man Rogosa Sharpe Broth; EMD Chemicals Inc., Darmstadt, Germany) [16]. *Escherichia coli* strains were grown at 37°C in low salt Luria Bertani (LB) medium with agitation. *L. lactis* subsp. *cremoris* MG1363 was grown in M17 broth (Quelab, Montreal, Canada) supplemented with 0.5% (w/v) glucose (GM17) incubated at 30°C. For *L. lactis* transformants, chloramphenicol was added at 5 μg/ml and 100 μg/ml ampicillin was added for *E. coli* transformants.

**Table 1:** **Bacterial strains and plasmids**

**Strain or plasmid**	**Relevant characteristic(s)**	**Source or reference**
Strains		
*L. rhamnosus* ATCC 9595	Low EPS-producing strain (116 mg/L)	ATCC^1^
*L. rhamnosus* RW-9595 M	High EPS-producing strain (1611 mg/L)	Denis Roy^2^
*L. lactis* subsp. *cremoris* MG1363	Plasmid free (Lac-Prt-)	[17]
*E. coli* NM522	Cloning host (*supE thi-1* ∆(*lac-proAB*) ∆(*mcrB–hsdSM*)*5* (rK– mK–) [F´ *proAB lacI* ^q^ *Z*∆*M15*])	Stratagene^1^
*E. coli* BL21(DE3)	Expression host (B F^−^, *omp*T, *hsd*S (r_B_^−^, m_B_^−^), *gal*, *dcm*)	EMD Chemicals
*E. coli* C41(DE3)	Expression host derived from BL21	[18]
Plasmids		
pMG36CT	Cm, 3.7 kb, pWV01 replicon	
pMG36EB	pMG36CT containing 1.6 kb *Xba*I-*Aat*II PCR amplicon (*wze* and *wzb*) from RW-9595 M	This study
pDWTEB	pMG36EB containing 923 bp *Sac*I-*Xba*I PCR amplicon (wild type *wzd*) from RW-9595 M	This study
pDY33FEB	pMG36EB containing 923 bp *Sac*I-*Xba*I PCR amplicon (Y33F mutated *wzd*) from RW-9595 M	This study
pDY44FEB	pMG36EB containing 923 bp *Sac*I-*Xba*I PCR amplicon (Y44F mutated *wzd*) from RW-9595 M	This study
pDY77FEB	pMG36EB containing 923 bp *Sac*I-*Xba*I PCR amplicon (Y77F mutated *wzd*) from RW-9595 M	This study
pDY110FEB	pMG36EB containing 923 bp *Sac*I-*Xba*I PCR amplicon (Y110F mutated *wzd*) from RW-9595 M	This study
pDY114FEB	pMG36EB containing 923 bp *Sac*I-*Xba*I PCR amplicon (Y114F mutated *wzd*) from RW-9595 M	This study
pDY124FEB	pMG36EB containing 923 bp *Sac*I-*Xba*I PCR amplicon (Y124F mutated *wzd*) from RW-9595 M	This study
pDY134FEB	pMG36EB containing 923 bp *Sac*I-*Xba*I PCR amplicon (Y134F mutated *wzd*) from RW-9595 M	This study
pDY141FEB	pMG36EB containing 923 bp *Sac*I-*Xba*I PCR amplicon (Y141F mutated *wzd*) from RW-9595 M	This study
pDY266FEB	pMG36EB containing 923 bp *Sac*I-*Xba*I PCR amplicon (Y266F mutated *wzd*) from RW-9595 M	This study
pQE30	His-tag fusion protein expression vector; Cm^r^, Am^r^	Qiagen^1^
pQE31	His-tag fusion protein expression vector; Cm^r^, Am^r^	Qiagen^1^
pGEX-6P-3	GST fusion protein expression vector	Amersham Biosciences^1^
pGSTWze	779 bp digested PCR fragment (*wze*) cloned into the *Bam*HI-*Xho*I site of pGEX-6P-3	GST-tag Wze; This study
pQEWzd	912 bp *Bam*HI/*Xho*I digested PCR fragment (*wzd*) cloned into the *Bam*HI-*Sal*I site of pQE30	His-tag Wzd; This study
pQEWze	819 bp digested PCR fragment (*wze*) cloned into the *Bam*HI-*Hin*dIII site of pQE31	native Wze; This study
pQEWzb	805 bp *Sst*I-*Kpn*I digested fragment (*wzb*) from pCRB cloned into the *Sst*I-*Kpn*I site of pQE31	native Wzb; This study

^1^ATCC (American Type Culture Collection, Manassas, VA, USA); EMD chemicals (Gibbstown, USA); Qiagen S.A. (Courtaboeuf, France); Stratagene (LaJolla, CA, USA); Amersham Biosciences (Orsay, France).

^2^Original source: Denis Roy, INAF, Université Laval. [19].

### Plasmid construction and verification

Plasmids used in this study are listed in Table 1 and oligonucleotide primers used are listed in Table 2. PCR was performed using standard conditions [20] with *Taq* Polymerase (Feldan-bio, Québec, Canada) and the primers listed in Table 1 specific for the *wzd*, *wze* and *wzb* sequences from *L. rhamnosus* strain ATCC 9595 [GenBank: AY659976] and RW-9595 M [GenBank: AY659979] [3]. For studying *in vitro* protein interactions, the amplicons were ligated to the vectors pQE30 and pGEX-6-P and *E. coli* strains were transformed with the resulting recombinant plasmids by a standard electroporation procedure [21].

**Table 2:** **Oligonucleotide primers**

**Primer** ^**1**^	**Sequence (5’ to 3’)**	**Gene target**
WzdRSacI	CGg agc tcA AGA GCA AAT TGA CCT TGC AC	*wzd*
WzdFXbaI	CGt cta gaT ACT TAA ACG CGT CTC CGG CTT CG	*wzd*
WzeRXbaI	GCG tct aga TTG AGG AGA AAA AAC ATG AAT TTT TC	*wze*
WzeF-Wzb	ATA CTA TCT AAG CTC AAT ACT TAA ACG CGT CTC CGG	*wze*
WzbR-Wze	ATA AAT AGC ATG CCT TAG ATA GTA TTG GAA GGG GAA C	*wzb*
WzbFAatII	CGg acg tcA TGA AAT TAG CAC TCG CAC AAC C	*wzb*
WzdSBamI	CGg gat ccA TTG ACC TTG CAC GAC TTT GG	*wzd*
WzdRXhoI	CCG ctc gag TAC TTA AAC GCG TCT CCG GC	*wzd*
WzeSBamI	CGg gat ccT CAT TAG AGA AAA TTT TGC ATA GAC	*wze*
WzeRXhoI	CCG ctc gag GAC AGT TAG AAG CGC ATG CT	*wze*
WzbSEcoI	Gga att cGA TTG ATG TGC ATT GCC ATA TG	*wzb*
WzbRXhoI	CCG ctc gag TAC CTT AAT ACC GCG ACA ACA AAC	*wzb*
WzeRhamS	CGg gat cca GAC GCG TTT AAG TAT TGA GGA G	*wze*
WzeRhamR	CCC aag ctt TGA CAG TTA GAA GCG CAT GC	*wze*

^1^Restriction sites in primers are indicated in lower case letters.

*L. lactis* subsp. *cremoris* MG1363 was used as host strain for the Wzy-dependent polymerization complex from *L. rhamnosus* RW-9595 M. A total of ten plasmids were constructed for expressing Wze and Wzb in conjunction with the wild type or nine mutated versions of the *wzd* gene in *L. lactis* subsp. *cremoris* MG1363 (Table 1). First, the two genes *wze* and *wzb* were amplified with primers wzeRXbaI/wzeF-wzb and wzbR-wze/wzbFAatII for the first PCR, and two amplicons were combined by PCR using wzeRXbaI as reverse primer and wzbFAatII as forward primer. The combined gene amplicon was cloned into pMG36CT to form pMG36EB. Nine separate tyrosine mutations to phenylalanine in the nucleotide sequence of *wzd* from RW-9595 M were constructed by gene synthesis (GenScript, USA). Each *Δwzd* gene was amplified and then cloned into pMG36EB (pMG36CT + *wze,wzb)* and the resulting recombinant plasmids were separately transformed in *L. lactis* using the previously-published electroporation procedure [22]. The inserts of all final constructs in each host strain were confirmed by sequencing with an ABI Prism 3100 apparatus.

### Production and purification of native and fusion proteins

An overnight *E. coli* culture was diluted 100-fold with fresh LB broth supplemented with ampicillin, which was incubated at 37°C with shaking until A_600_ reached 0.5 to 0.6 (equivalent to 1 to 2 x 10^8^ CFU). Induction was initiated by adding IPTG to 1 mM and incubation continued for 3 h with shaking at 37°C. Cells were centrifuged at 18000 X *g* for 20 min at 4°C and the pellet was suspended in 15 ml STE buffer (10 mM Tris–HCl, pH 8.0, 150 mM NaCl and 1 mM EDTA) containing 200 μg/ml lysozyme. After 10 min incubation on ice, the cells were sonicated (40 W, 5 min) and then centrifuged at 18000 X *g* for 20 min at 4°C [23]. The cell pellet was suspended in 2 ml ST buffer (50 mM Tris, 300 mM NaCl, 5 mM ZnCl_2_ and 20 mM β-mercaptoethanol) containing 10% sarcosyl and incubated overnight at 4°C [24]. The resulting suspensions of proteins were diluted 10 times with PBS (phosphate-buffered saline). The 20 ml suspension of His_6_-Wzd was added to 1 ml Ni^2+^ nitrilotriacetic acid (Ni^2+^-NTA) agarose resin (Qiagen) with 20 mM imidazole. Batch binding was carried out overnight (18 h) with gentle stirring at 4°C. For GST-Wze, glutathione sepharose 4B (GE Healthcare Life Sciences) was utilized for purification. When needed to ensure complete dephosphorylation, a 0.3 ml aliquot of resin-bound His_6_-Wzd was treated with 600 U YOP (*Yersinia* tyrosine phosphatase, New England BioLabs) at 30°C for 3 h, then inactivated by heat (65°C for 1 h). YOP-treated and untreated aliquots (0.3 ml) were then transferred to columns and washed 3 times with PBS containing 20 mM imidazole.

For proteins without any tag (Wzb and Wze), the cell pellet from 100 ml of induced culture was suspended in 15 mL PBS (pH 7.3) then incubated for 20 minutes at 25°C with 200 μg/ml lysozyme. Lysis was completed by two passages on a French Press (138 MPa, 6°C) followed by centrifugation at 18000 X *g* 15 min, then the pellet was washed three times with PBS containing 5% Triton X-100 and 2 M urea, followed by two washes with PBS. The washed pellet was suspended in 5 ml PBS containing 2% sarcosyl then proteins were precipitated by adding 7.5 ml acetone followed by centrifugation for 30 min at 18000 X *g*. Sarcosyl was eliminated by two washes with 2.5 ml 70% ethanol then the pellet was suspended in 1 ml PBS containing 5 mM MgCl_2_ and 1 mM DTT.

A volume of 15 ml of *L. lactis* subsp. *cremoris* cells grown to 5 × 10^8^ CFU/ml (A_600_ of 0.65) in GM17 was centrifuged at 20000 X g for 10 min at 4°C. The pellet was suspended in 1 ml TE buffer (10 mM Tris (pH 8.0) and 0.1 mM EDTA) and mechanically disrupted in a mini beadbeater (BioSpec, Bartlesville, USA) with 1 g of 0.1 mm dia. glass beads (BioSpec, Bartlesville, USA) by two 30 s treatments with 1 min cooling on ice between two treatments [11]. Twenty microliters of cell extract was separated by SDS-PAGE.

### Protein-protein interaction assay

The *in vitro* protein interaction was carried out by His-pull down assay. Either 1 ml of purified Wze or 10 ml of diluted cell lysate of GST-Wze with 20 mM imidazole were added to an aliquot (300 μl) of the resin-conjugated His_6_-Wzd and incubated overnight (18 h) with stirring at 4°C. In duplicate samples, 10 mM ATP (Sigma) was added before the overnight incubation. The protein mixtures were then transferred to a column and washed with PBS containing 20 mM imidazole. In duplicate wash solutions, either 200 μM or 10 mM ATP was added. When necessary, elution of bound proteins was carried out with 1 M imidazole.

### Western immunoblotting

Proteins separated by SDS-PAGE were transferred by semi-dry electroblotting onto polyvinylidene difluoride membranes in triplicate to detect proteins with three different antibodies. Phosphotyrosine protein detection was carried out with primary antibodies: the membrane blots were incubated overnight at 4°C using a 1:1000 dilution of mouse monoclonal IgG anti-phosphotyrosine 4G10 antibody (Millipore). Detection of tagged protein with His_6_ or GST was carried out with primary antibodies using a 1:1000 dilution of mouse monoclonal IgG_2ak_ anti-histidine tagged antibody (Millipore) or mouse monoclonal IgG anti-GST tag antibody (Millipore), respectively. All membranes were incubated for 1 h with 1:20000 dilution of secondary antibody (goat anti mouse IgG) coupled with horseradish peroxidase (HRP) (Millipore). Supersignal substrate (Thermo scientific) was used for HRP detection and visualised by exposure to Amersham Hyperfilm ECL (GE).

### Ethics statement

No human subjects, human material, or human data were used in this study.

## Results and discussion

Polymerization must be strictly controlled with respect to precursor availability and the energy necessary to form glycosidic bonds as well as to transport the units out of the cell, and finally to attach the polymers to the cell in the form of capsular material or to release them. Significant advancement has been made in understanding some interactions among components of the biosynthetic complex, but further work is needed to elucidate how these interactions promote the assembly of polysaccharides on the cell surface. In *L. rhamnosus*, three proteins may play a role in the control of EPS production, namely Wzb (phosphatase), Wzd (tyrosine protein kinase transmembrane modulator) and Wze (phosphotyrosine kinase). Details of comparative sequence analysis of Wzb, Wzd and Wze can be found in the Additional file 1 (Figure S1 and S2). In this study, the three genes from *L. rhamnosus* strain RW-9595 M were transferred to *L. lactis* subsp. *cremoris* MG1363 in order to discover the phosphorylation state of the three proteins *in vivo* in a Gram-positive expression system, as well as to demonstrate the importance of individual tyrosine residues to total Wzd phosphorylation and to the phosphorylation state of Wze. In order to examine protein interactions *in vitro* and their effect on phosphorylation, the three genes *wzb*, *wzd* and *wze* were expressed in *E. coli* to obtain tagged and untagged proteins.

### Tyrosine phosphorylation of nine tyrosine-mutated Wzd proteins in *L. lactis*

**Figure 1:**
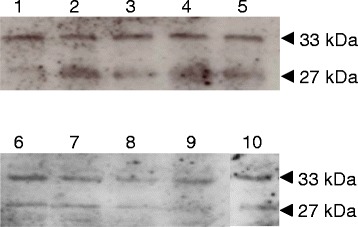
**Immunoblot showing tyrosine phosphorylation of wild-type Wzd, ΔWzd and Wze proteins expressed in** ***L. lactis*** **subsp.** ***cremoris*** **MG1363** ***.*** Detection of tyrosine phosphorylation of Wze (27 kDa) along with wild type Wzd (33 kDa) or nine separate tyrosine mutated Wzd versions (33 kDa) expressed in *L. lactis* subsp. *cremoris* MG1363 with plasmids pDY33FEB (lane 1), pDY44FEB (lane 2), pDY77FEB (lane 3), pDY110FEB (lane 4), pDY114FEB (lane 5), pDY124FEB (lane 6), pDY134FEB (lane 7), pDY266FEB (lane 8), pDY141FEB (lane 9), pDWTEB (lane 10). Cell lysates of *L. lactis* subsp. *cremoris* MG1363 transformants were separated by 12% SDS-PAGE and tyrosine phosphorylated proteins were probed with mouse monoclonal anti-phosphotyrosine antibody.

Nine mutated *wzd* genes changing a different tyrosine codon to phenylalanine were expressed individually in concert with *wze* and *wzb* from *L. rhamnosus* RW-9595 M in *L. lactis* subsp. *cremoris* MG1363 carrying one of the nine constructed plasmids (Table 1). As negative controls, there was no phosphorylated protein when extracts of *L. lactis* subsp. *cremoris* MG1363 with or without plasmid pMG36CT were probed with the anti-phosphotyrosine antibody (data not shown). All nine separate tyrosine mutated Wzd proteins (33 kDa) were phosphorylated (Figure 1). For six mutants (Figure 1, Lanes 1 to 6), band density was approximately equivalent to the wild-type Wzd (ratio of 1, as determined by ImageJ densitometry analysis). Two mutant proteins had slightly less band density than wild-type Wzd (Figure 1, Lane 10) with ratios of 0.9 (Wzd mutant Y134F; Lane 7) and 0.78 (Wzd mutant Y141F; Lane 9). The WzdY266F protein had the lowest ratio of 0.54 (Figure 1, lane 8) with respect to wild-type Wzd. Wze (27 kDa) was also phosphorylated when co-expressed with each of the nine mutated Wzd proteins in *L. lactis* subsp. *cremoris* MG1363 (Figure 1), albeit to a lower level when Wzd carried mutations Y33F, Y266F and Y241F (Figure 1, Lanes 1, 8 and 9).

### Expression and purification of tagged and untagged proteins

**Figure 2:**
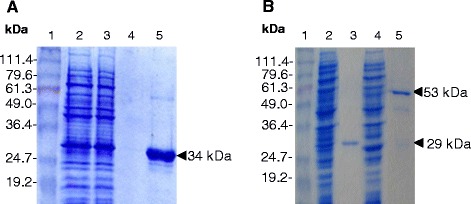
**SDS-PAGE of IPTG-induced cell extracts and purified Wzd, Wzb and GST-Wze. (A)** 34 kDa His_6_-Wzd fusion protein purification by affinity chromatography from *E. coli* C41(DE3) (pQEWzd) IPTG-induced culture lysate. Lane 2: The flow through contains proteins not retained by the Ni^2+^ column. Lanes 3 and 4: fractions from first and last column washes, respectively; Lane 5: fraction from the elution step. **(B)** Wzb and GST-Wze purified from IPTG-induced culture lysates of *E. coli* NM522 (pQEWzb) and *E. coli* BL21(DE3) (pGSTWze). Lanes 2 and 4: lysed culture supernatant; Lanes 3 and 5: purified Wzb (29 kDa) and GST-Wze (53 kDa) proteins. Lane 1: BenchMark Prestained Protein Ladder (Invitrogen).

Overexpression of the 34 kDa His_6_-tagged Wzd protein from the *wzd* gene of *L. rhamnosus* ATCC 9595 was achieved in two *E. coli* strains, namely BL21(DE3) or C41(DE3) carrying pQEWzd (Figure 2A). Untagged Wzb and Wze proteins of the predicted molecular mass were obtained in *E. coli* strain NM522 carrying either pQEWzb (Figure 1B) or pQEWze. GST-tagged Wze protein was obtained using both *E. coli* strains C41(DE3) or BL21(DE3) carrying pGSTWze (Figure 2B).

### Tyrosine phosphorylation of His_6_-Wzd and GST-Wze expressed in *E. coli*

**Figure 3:**
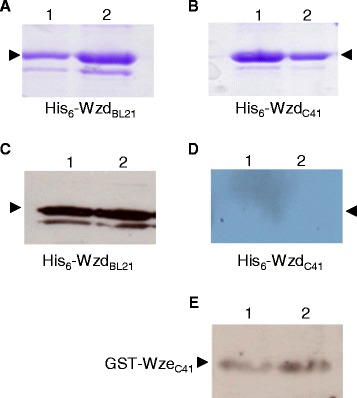
**Western immunoblot of the phosphorylation state of His**_**6**_ **-Wzd and GST-Wze proteins expressed in** ***E. coli.*** Tyrosine phosphorylation of His_6_-Wzd and GST-Wze was detected by using Western immunoblotting with a mouse monoclonal antibody. Coomassie stained 12% SDS-PAGE gel of His_6_-Wzd expressed in *E. coli* BL21(DE3) **(A)** and C41(DE3) **(B)**: incubated with 10 mM ATP (Lane 1) and without ATP (Lane 2). **(C)** Detection of tyrosine phosphorylated His_6_-Wzd expressed in *E. coli* BL21(DE3) and incubated with 10 mM ATP (Lane 1) and without ATP (Lane 2). **(D)** Absence of detection of tyrosine phosphorylated His_6_-Wzd expressed in *E. coli* C41(DE3) and incubated with 10 mM ATP (Lane 1) and without ATP (Lane 2). **(E)** Detection of tyrosine phosphorylated GST-Wze expressed in *E. coli* C41(DE3) incubated with 10 mM ATP (Lane 1) and without ATP (Lane 2).

The phosphorylation state of the expressed proteins was verified prior to testing protein interactions. To detect any autophosphorylation, proteins were incubated with or without ATP. As controls, cell lysates of the host strains BL21(DE3) and C41(DE3) carrying the vector plasmids did not show any tyrosine phosphorylated proteins, with or without added ATP (data not shown). In the absence of added ATP, the His_6_-Wzd purified from *E. coli* BL21(DE3) was recognized by the anti-phosphotyrosine antibody, which shows tyrosine phosphorylation (Figure 3C). On the other hand, when purified after expression by strain C41(DE3), the similar amount of His_6_-Wzd was not phosphorylated after incubation either with or without ATP (Figure 3D), even though the protein was detected at by Western blot using the anti-His-tag antibody (data not shown). Under these conditions, the amount of phosphorylated His_6_-Wzd protein may be below the detection level of our assay.

The presence of GST-Wze was confirmed by anti-GST antibody and, as a control, GST expressed alone was not detected by the phosphotyrosine antibody (data not shown). The anti-phosphotyrosine antibody did detect phosphorylated GST-Wze in cell lysates of *E. coli* C41(DE3) pGSTWze (Figure 3E) at the same level as in cell lysates of *E. coli* BL21(DE3) pGSTWze (data not shown) for the same amount of protein. The level of phosphorylated GST-Wze protein did not differ in the presence or absence of 10 mM ATP. However, the untagged native Wze protein was not phosphorylated when produced by *E. coli* strains NM522 or BL21(DE3), whether or not ATP was present.

While GST-Wze was slightly phosphorylated in all *E. coli* host strains tested, the tyrosine phosphorylation of His_6_-Wzd depended on the strain of *E. coli* used for expression. The initial level of phosphorylation of these two proteins did not change when ATP was added. The cause of the difference between strain BL21(DE3) and its derivative mutant C41(DE3) has been attributed to a lower amount of T7 RNA polymerase in strain C41(DE3), which reduces transcription levels of cloned genes, actually leading to a more stable over-expression of recombinant proteins by strain C41(DE3) [25]. Phosphorylation of His_6_-Wzd may conceivably be carried out by a PTK of *E. coli* BL21(DE3), as was observed in studies of other proteins [26]. In our study, no other tyrosine-phosphorylated proteins were detected in the *E. coli* host strains used. UDP-glucose dehydrogenase YwqF from *B. subtilis* was phosphorylated by PTK Wzc from *E. coli*, showing that Gram-negative PTK is capable of phosphorylating proteins from Gram-positive species. Wze possesses a tyrosine in a similar position as the tyrosine 569 implicated in autophosphorylation of Wzc (PTK) in *E. coli* [27]. The autophosphorylation of tyrosine 569 did not occur without the N-terminal domain of Wzc, which is similar to the tyrosine kinase transmembrane modulator protein from Gram positive bacteria [28]. Thus, initial phosphorylation of GST-Wze implies that the presence of the GST tag may play a role as the modulator protein or that a protein of *E. coli* carries out tyrosine phosphorylation. Although serine phosphorylation of GST has been demonstrated previously [29], no tyrosine phosphorylation of GST itself was detected in our study (data not shown). GST may, however, possibly be phosphorylated by Wze. Nevertheless, there is no evidence of higher phosphorylation when GST-Wze is expressed alone, although the kinase is proposed to have autophosphorylation activity. This means that the Wzd modulator protein is required for higher levels of phosphorylation of the Wze tyrosine kinase, as described by Bender & Yother [8]. To our knowledge, no previous study has detected the phosphorylation of the tyrosine kinase transmembrane modulator protein in any Gram-positive bacterial species.

### Interaction of His_6_-Wzd with Wzb and Wze

**Figure 4:**
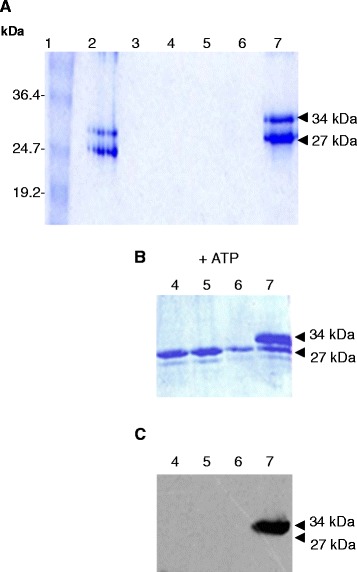
***In vitro*** **interaction assay between resin-bound His**_**6**_ **-Wzd**_**C41**_ **(34 kDa) and native Wze (27 kDa) proteins.** Coomassie-stained gel A and B: **(A)** Lane 1 contains the BenchMark Prestained Protein Ladder (Invitrogen). Lane 2: The two proteins were incubated together in a 1:1 ratio with the Ni^2+^ charged resin for 4 h at 16°C in 2.5 ml buffer B1 containing 10 mM Tris–HCl pH 7.5, 100 mM NaH_2_PO_4_, 50 mM imidazole, 1% Triton, 5 mM MgCl_2_. Lanes 3 to 6: 5 ml wash fractions with buffer B1 in panels **A** and **B** (in panel **B**, 200 μM ATP was added to the wash in lanes 4 to 6). Lane 7: protein elution using 1 ml buffer containing 10 mM Tris–HCl pH 7.5, 100 mM NaH_2_PO_4_, 1 M imidazole, 1% Triton, 5 mM MgCl_2_, 2% sarcosyl. **(C)** Western blot of gel in panel **B**, detection was carried out using anti-phosphotyrosine antibody.

Untagged Wzb and Wze were not retained by the nickel-charged affinity resin itself (data not shown), so they could be tested for affinity to resin-bound His_6_-Wzd. Wzb was not retained by His_6_-Wzd after washing the resin (data not shown), suggesting that no stable interaction was present between these two proteins. After incubating resin-bound His_6_-Wzd_C41_ and Wze together, the resin was divided into two fractions for washing either with or without ATP. Wze was retained by His_6_-Wzd using the same washing conditions as with Wzb (Figure 4A). Without added ATP, the wash fraction did not contain any tyrosine-phosphorylated protein. However, when 200 μM ATP was added to the washing solution, Wze protein was released from resin-bound His_6_-Wzd in each of three successive washing steps (Figure 4B, lanes 4, 5 and 6). After eluting the remaining bound proteins from the same column, only a small quantity of non-phosphorylated Wze was associated with His_6_-Wzd (Figure 4B, Lane 7). Moreover, using the anti-phosphotyrosine antibody, the Wzd protein was revealed to be tyrosine-phosphorylated after washing with 200 μM ATP (Figure 4C).

**Figure 5:**
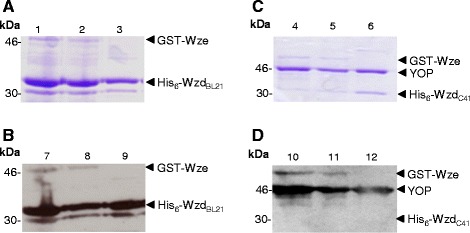
***In vitro*** **interaction assays between His**_**6**_ **-Wzd (34 kDa) and GST-Wze (53 kDa) proteins.** His_6_-Wzd bound to Ni^2+^ charged resin was incubated with the cell lysate of GST-Wze in the presence of 10 mM ATP (lanes 1, 4, 7 and 10) or absence of ATP (lanes 2, 5, 8 and 11). Lanes 3, 6, 9 and 12 contain native His_6_-Wzd alone on the Ni^2+^ resin. Interaction of GST-Wze with His_6_-Wzd expressed by *E. coli* BL21(DE3): **(A)** Coomassie stained 12% SDS-PAGE gel and **(B)** Western blot probed with mouse monoclonal anti-phosphotyrosine antibody. Interaction of GST-Wze with resin-bound His_6_-Wzd expressed by *E. coli* C41(DE3) and treated with YOP (*Yersinia* tyrosine phosphatase): **(C)** Coomassie stained 12% SDS-PAGE gel and **(D)** Western blot probed with mouse monoclonal anti-phosphotyrosine antibody.

GST-Wze alone also did not bind to the Ni^2+^ resin in the absence of His_6_-Wzd (data not shown). A cell lysate of *E. coli* C41(DE3) pGSTWze was passed through a column containing purified His_6_-Wzd expressed by either *E. coli* BL21(DE3) (His_6_-Wzd_BL21_) or C41(DE3) (His_6_-Wzd_C41_) bound to the Ni^2+^ resin (Figure 5). GST-Wze was retained by His_6_-Wzd_BL21_, both in the presence and absence of 10 mM ATP during incubation and washing (Figure 5A). In the presence of ATP, there was no discernible change of tyrosine phosphorylation of His_6_-Wzd_BL21_ after the interaction (Figure 5B). GST-Wze was also retained by dephosphorylated His_6_-Wzd_C41_, in the presence and absence of 10 mM ATP while YOP was retained as well (Figure 5C). YOP treatment did not affect protein migration and was not found in the wash fraction. In the presence or absence of ATP, His_6_-Wzd_C41_ on the resin with GST-Wze was not phosphorylated (Figure 5D). Bound GST-Wze showed slightly more phosphorylated protein in the presence of ATP, but the difference does not appear significant and there was no discernible change in phosphorylation either before or after the interaction with His_6_-Wzd. The identical result was obtained using His_6_-Wzd_C41_ without YOP treatment and GST-Wze.

**Figure 6:**
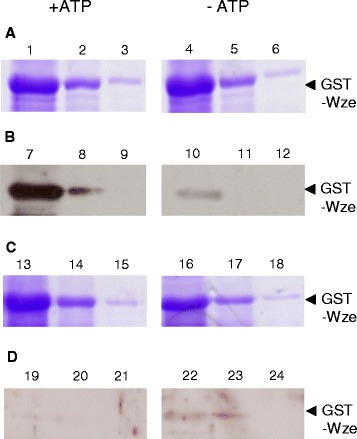
**Detection of tyrosine phosphorylated GST-Wze released in the wash fractions.** GST-Wze in wash fractions in the presence of 10 mM ATP (1–3 and 7–9) and absence of ATP (4–6 and 10–12) from interaction of GST-Wze with resin-bound His_6_-Wzd expressed by *E. coli* BL21(DE3): **(A)** Coomassie stained 12% SDS-PAGE gel and **(B)** Western blot probed with mouse monoclonal anti-phosphotyrosine antibody. GST-Wze in wash fractions in the presence of 10 mM ATP (13–15 and 19–21) and absence of ATP (16–18 and 22–24) from interaction of GST-Wze with His_6_-Wzd expressed by *E. coli* C41: **(C)** Coomassie stained 12% SDS-PAGE gel and **(D)** Western blot probed with mouse monoclonal anti-phosphotyrosine antibody.

In contrast, the phosphorylation state of GST-Wze in the wash fractions was very different from that bound to His_6_-Wzd on the Ni^2+^ resin. For the same amount of protein (Figure 6A, lanes 1, 2 and 3), GST-Wze was found in the wash fraction with a high phosphorylation level (Figure 6B, lanes 7, 8 and 9), after interacting with His_6_-Wzd_BL21_ in the presence of ATP. In the absence of ATP, the phosphorylated GST-Wze signal in the wash fraction was much lower (Figure 6B, lanes 10 to 12) for the same amount of protein (Figure 6A, lanes 4 to 6). There appears to be some phosphorylated GST-Wze retained by His_6_-Wzd, but the highly phosphorylated GST-Wze did not bind strongly to Wzd. After interacting with His_6_-Wzd_C41_, GST-Wze was washed out of the resin, and the low level phosphorylation does not appear to significantly differ in the presence or absence of ATP (Figure 6C and D).

The results of this study reveal that no strong protein association was formed between Wzb and Wzd, while Wze interacts with phosphorylated as well as non-phosphorylated Wzd. When only slightly phosphorylated, GST-Wze did not affect the phosphorylation of Wzd, while Wzd was phosphorylated after interaction with non-phosphorylated Wze. This result shows that non-phosphorylated Wze is able to phosphorylate Wzd. In the presence of 10 mM ATP, the unbound Wze was highly phosphorylated after interaction with phosphorylated Wzd, whereas Wze released from interaction with non-phosphorylated Wzd was not changed. The phosphorylation state of Wze retained with Wzd on the resin did not change after the interaction, regardless of the phosphorylation state of Wzd. This means that phosphorylated Wzd and ATP are necessary to attain a higher phosphorylation state of Wze and phosphorylated Wze does not remain bound in close association with phosphorylated Wzd.

Cefalo *et al.* [30] detected the interaction between Wzd (designated a transmembrane activator protein) and Wze (tyrosine kinase) in *S. thermophilus* and previous work done in *S. pneumoniae* [10] showed that CpsC/CpsD (modulator/kinase) complex formation is necessary to detect tyrosine phosphorylated CpsD (tyrosine kinase). Our work shows evidence for this complex formation in *L. rhamnosus* and for tyrosine phosphorylation of both the kinase as well as the modulator protein. The results also support the role of Wzd as a modulator of the phosphorylation of the tyrosine kinase. EPS was not produced after deletion of EpsC (modulator) in *S. thermophilus* CNRZ1066 [12]. The Wzd modulator protein from *L. rhamnosus* could be involved in the production of EPS as proposed for EpsC. Indeed, several mutations occurring in the CpsC transmembrane modulator of a number of *S. pneumoniae* strains lead to defective cell wall attachment of CPS [6]. These include two tyrosine mutations in the N-terminal region of CpsC (Y40C and Y83F) associated with a mucoid colony phenotype. In comparison with the *L. rhamnosus* Wzd sequence, equivalent tyrosine residues can be found in similar positions (Y33 and Y77) (see Additional file 1: Figure S2).

Wzd from *L. rhamnosus* has nine tyrosine residues and the last one (Tyr266) is located in the C-terminal cytoplasmic domain. The *wzd, wze* and *wzb* genes from *L. rhamnosus* RW-9595 M were thus expressed in *L. lactis* subsp. *cremoris* MG1363 in order to verify which tyrosine residues could be involved in Wzd phosphorylation and whether Wze is then phosphorylated *in vivo*. While each of the nine separate tyrosine mutations led to Wzd phosphorylation, mutation of the C-terminal tyrosine (Tyr266) showed slightly weaker Wzd phosphorylation than most of the other eight mutations. This suggests that more than one tyrosine residue participates in Wzd phosphorylation, although Tyr266 appears to be slightly more important. In all cases, Wze was phosphorylated, although the phosphorylation levels of Wze were slightly lower when Wzd was mutated in positions Y33 (N-terminus), Y141 or Y266 (C-terminus). A previous study observed that the C-terminal cytoplasmic domain of CapA (transmembrane modulator) was essential for phosphorylation of CapB (kinase) in *Staphylococcus aureus* [31]. Tocilj *et al.* [32] proposed a role for the C-terminal Y191 tyrosine residue in alpha helix stabilization of the modulator, but no phosphorylation was proposed. Therefore, one or more of the mutated residues may be important for proper folding and membrane insertion of Wzd to allow good interaction with Wze, and thus facilitate the auto- or trans-phosphorylation of Wze.

In *S. pneumoniae* D39*,* when the *cps2C* (transmembrane modulator) was deleted, no capsule was detected, but low molecular weight products could be observed [9]. Defects in cell wall attachment were found when point mutations were introduced into *cps2C*, while some mutants were able to maintain wild type levels of total CPS production, including the Y82C mutation [9]. EPS were not produced at all when the equivalent modulator encoded by *epsA* was deleted in *L. lactis* [11]. Cps2C from *S. pneumoniae* strain D39 contains 5 tyrosine residues while Cps2C from strain Rx1-19 F contains only 3, all located within the first 100 N-terminal residues, as does the modulator of *S. thermophilus* and *S. aureus*. No tyrosine residue appears conserved among all 13 putative PCP2b sequences compared from staphylococci, streptococci, lactococci and lactobacilli, although the tyrosine positioned right after the first transmembrane domain is found in 12 out of the 13 sequences aligned. This indicates that the precise function of PCP2b could differ among species. The lactobacilli PCP2b sequences contain a supplementary stretch of 46 amino acids including 3 to 5 tyrosine residues that are not found in sequences from streptococci, lactococci and staphylococci. Wzd (*L. rhamnosus*), EpsA (*L. lactis* subsp. *lactis* or *cremoris*) and EpsB (*L. johnsonii*) have a total of 9, 7 and 6 tyrosine residues, respectively. The genera *Lactobacillus* and *Lactococcus* have common conserved tyrosines (both Y44 in the N-terminus and Y266 in the C-terminal region) in their modulator protein sequences. These conserved tyrosines, especially the tyrosine located near the C-terminal transmembrane region, may be associated with polymer extension and release instead of attachment. An equivalent C-terminal tyrosine 266 is absent from the CpsC sequences of *S. pneumoniae,* and *S. thermophilus* as well as the CapA sequence of *S. aureus*. Future work will need to focus on how these differences impact the production of capsular polysaccharides versus the production of released high molecular weight EPS.

## Conclusion

**Figure 7:**
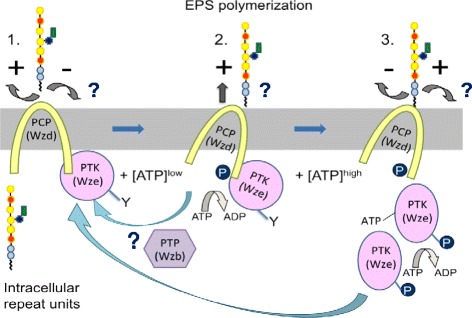
**Model proposed for the tyrosine switch controlling EPS polymerization and attachment. 1.** Non-phosphorylated Wzd and Wze interact by forming a complex leading to release of EPS (+) rather than attachment (−). **2.** In the presence of ATP, Wze phosphorylates Wzd, allowing chain elongation by the polymerase (+). This phosphorylation of Wzd destabilizes the protein interaction with Wze, allowing Wze to either bind with other non-phosphorylated Wzd proteins, or undergo auto- or trans-transphosphorylation. **3.** A transitory interaction between phosphor-rylated Wzd and Wze in the presence of ATP is necessary for the phosphorylation of Wze, possibly allowing the attachment of polysaccharide to the cell wall (+). Phosphatase activity of Wzb for dephosphorylating Wzd and Wze would return the cycle to the non-phosphorylated Wzd/Wze complex that allows release of polysaccharide polymers (+). Question marks indicate proposed steps.

Our study shows that the activity of Wzd is also modulated through tyrosine phosphorylation of more than one tyrosine residue, allowing the phosphorylation of Wze. This new information suggests that further modification of the model for the control of EPS elongation can be proposed (Figure 7). The non-phosphorylated complex may allow polysaccharides to be released instead of attached to the cell wall. When Wze phosphorylates Wzd, interaction of Wzd with the polymerase may promote polysaccharide elongation [14]. Destabilization of the protein interaction between Wzd and Wze liberates Wze to interact with other non-phosphorylated Wzd or Wze proteins. When Wzd is phosphorylated and ATP is present, a transitory interaction allows Wze to autophosphorylate or transphosphorylate other Wze proteins, possibly slowing polymerization and leading to the attachment of polysaccharide to the cell wall if a ligase is present.

*L. rhamnosus* ATCC 9595 and RW-9595 M produce exopolysaccharides through a polymerase-dependent mechanism. Future experiments will focus on the role of the Wzb phosphatase in complex formation or dissociation, the relationship between the Wzd/Wze complex formation and EPS polymerization as well as the interaction of other proteins with this complex, including interactions of the polymerization complex with the proteins involved in synthesizing and transporting the repeat units. This approach will contribute to determining their function in modulating the biosynthesis of exopolysaccharides by *L. rhamnosus*.

## Additional file

Additional file 1:
**Comparative sequence analysis of Wzb, Wzd and Wze.** **Figure S1.** Comparison of transmembrane helix score plot of Wzd **(A)** from *L. rhamnosus* and CpsC19f **(B)** [GenBank: U09239]. **Figure S2.** Alignment of polysaccharide co-polymerases of thePCP2b subclass.

## Abbreviations

EPS: Exopolysaccharide
CPS: Capsular polysaccharide
PTP: Protein tyrosine phosphatase
PTK: Protein tyrosine kinase
PCP: Polysaccharide co-polymerase
*L rhamnosus*: *Lactobacillus rhamnosus*
*S pneumonia*: *Streptococcus pneumonia*
*S thermophilus*: *Streptococcus thermophilus*
*L lactis*: *Lactococcus lactis*
*E coli*: *Escherichia coli*
