# Supplementary material for: A tyrosine phosphorylation switch controls the interaction between the transmembrane modulator protein Wzd and the tyrosine kinase Wze of Lactobacillus rhamnosus
Source: BMC Microbiol. 2015 Feb 21;15:40. doi: 10.1186/s12866-015-0371-2 (PMC4340800; doi:10.1186/s12866-015-0371-2)
Supplement: Additional file 1: — Comparative sequence analysis of Wzb, Wzd and Wze. Figure S1. Comparison of transmembrane helix score plot of Wzd (A) from L. rhamnosus and CpsC19f (B) [GenBank: U09239]. Figure S2. Alignment of polysaccharide co-polymerases of thePCP2b subclass. [file 12866_2015_371_MOESM1_ESM.pdf]

Supplementary Material

Comparative sequence analysis of Wzb, Wzd and Wze

Among four strains of *Lb. rhamnosus* (ATCC 9595, RW-9595M, R, RW-6541M), Wzb is 100 % identical for three strains while a one amino acid change (T53 to A) occurs for the Wzb of strain RW-6541M [GenBank: AY659977] [1]. Phosphoesterase activity was predicted for Wzb, based on alignment of deduced amino acid sequences with PHP superfamily members (PHP domain, PF02811). Four conserved motifs of the PHP (polymerase and histidinol phosphatase) domain identified by Aravind & Koonin [2] were located in the amino acid sequence of Wzb. These motifs consist of conserved histidine and aspartic acid residues. Previous work [3] has demonstrated the phosphatase activity of Wzb.

Wzd is a 34 kDa protein coded by the 915 bp *wzd* gene located at the beginning of the gene clusters coding for EPS biosynthesis in *Lb. rhamnosus* ATCC 9595, just downstream of promoter P1 [1]. Hydrophobicity analysis predicts two transmembrane domains (amino acids 18 to 36 and 219 to 238, TMpred^{TM}; [4]; http://www.ch.embnet.org/software/ TMPRED_form.html), which were confirmed by SAPS (Statistical Analysis of Protein Sequences; [5]: http://www.isrec.isb-sib.ch/software/SAPS_form.html). In addition, this protein contains a coiled-coil region (not a leucine zipper) in the C-terminal intracellular region (amino acids 263 to 290). Comparison of the hydrophobicity profile of Wzd with that of CpsC from *S. pneumoniae* Rx1-19F (Fig. S1) suggests the function of co-polymerase. Comparison with similar proteins from other bacterial genera showed that Wzd corresponds to the N-terminal region of Wzc et ExoP, respectively from *E. coli* [6] et *S. meliloti* [7] and is an ortholog of EpsC from *S. thermophilus* as well as EpsA from *L. lactis*. There are three classes of polysaccharide co-polymerases (PCPs). The PCP1 are

associated with LPS chain length regulation [8, 9]. The PCP2 is involved in the synthesis of high molecular weight polysaccharides, such as CPS and EPS. Both PCP1 and PCP2 type proteins are involved in the Wzy dependent mechanism of polysaccharide biosynthesis. The last group, PCP3, participates in the biosynthesis of CPS via a mechanism employing an ABC (ATP Binding Cassette) transporter. The PCP2 proteins are divided into two subgroups: PCP2a (Gram negative bacteria) and PCP2b (Gram positive bacteria). The PCP2b subgroup includes CpsC (co-polymerase) from *S. pneumoniae* [9], the PCPs from *Staphylococcus aureus*, *S. thermophilus* and *L. lactis* [10], along with PCP from *Lb. rhamnosus* ATCC 9595 and RW-9595M (Fig S2). The alignment shows that PCP2b sequences may be further divided into three subgroups (1-lactobacilli; 2-lactococci; 3-staphylocci/streptococci). PCP2b from lactococci and lactobacilli have one tyrosine residue after the trans-membrane domain in the C-terminal region and a potential coiled-coil region, which are absent from staphylococci and streptococci. Furthermore, the PCP2b sequences from lactobacilli can be differentiated from those of lactococci, staphylococci and streptococci by the presence of a 46 amino acid sequence containing five tyrosine residues that are highly conserved within four of the five *Lactobacillus* putative PCP2b proteins examined. The only tyrosine that is present in almost all of the PCP2b sequences examined (12 out of 13 sequences; Fig. S2) is the one located in the N-terminal region just following the first transmembrane sequence.

Wze is coded by the second gene of the locus responsible for EPS biosynthesis and is cotranscribed with *wzd*. The 753 bp *wze* gene codes for a 27 kDa protein which is predicted by TMpred^{TM} to be cytoplasmic due to the absence of any transmembrane sequences. Wze has an ATP-binding site consisting of a Walker A sequence ([AG]X_4GK[ST] where X is any amino acid and two Walker B sequences (hhhD, where h represents a hydrophobic amino acid) in the N-

terminus and a C-terminus containing a tyrosine-rich region. The Walker A site is considered a common structural motif for prokaryotic protein kinases [11]. Finally, Wze has a tyrosine residue similar to the tyrosine 569 which was described as being implicated in autophosphorylation of Wzc in *E. coli* [12]. Alignment of this sequence with proteins proposed to have a role in polysaccharide production suggests that Wze would function as a protein tyrosine kinase, orthologous to CpsD from *S. pneumoniae* Rx1-19F, to EpsD from *S. thermophilus* Sfi6 and EpsB from *L. lactis* subsp. *cremoris* [13]. In addition, Wze corresponds to the C-terminus of the Wzc protein from *E. coli* [6] as well as ExoP from *S. meliloti* [7].

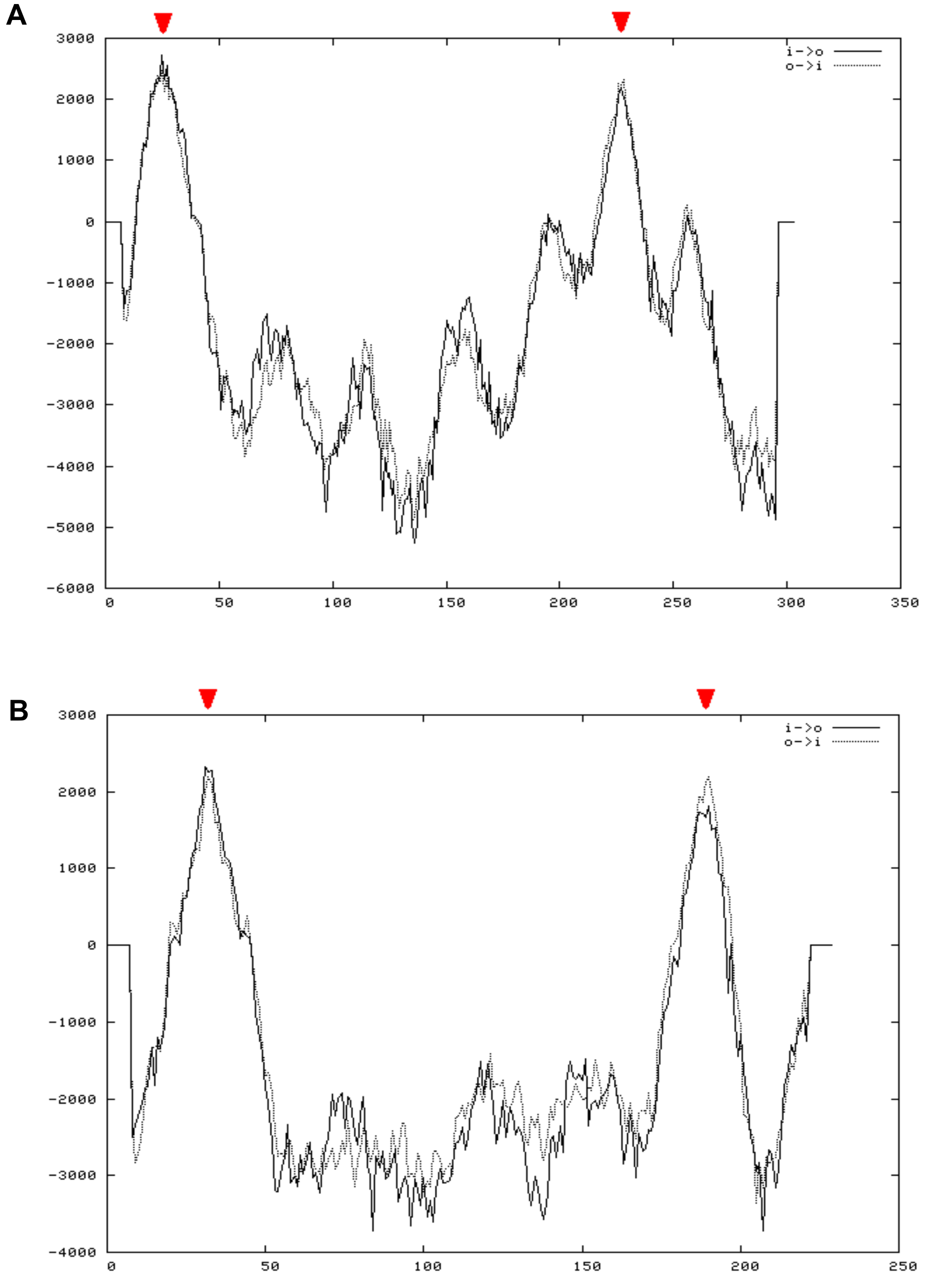

Figure S1. Comparison of transmembrane helix score plot of Wzd (A) from *L. rhamnosus* and CpsC19f (B) [GenBank: U09239].

Arrows indicate transmembrane domains (TMS1, 2). Transmembrane prediction was carried out with TMpred (http://www.ch.embnet.org/software/TMPRED_form.html). The preferred orientation is in-out for TMS1 and out-in for TMS2.

```
EpsA B40 --MQETQEQTIDLRGIFKIIRKRLGLILFSALIVTILGSIYTFFIASPVYTASTQLVVKL 58
EpsA     --MQETQEQTIDLRGIFKIIRKRLGLILFSALIVTILGSIYTFFIASPVYTASTQLVVKL 58
EpsA KF  --MQETQEQTIDLRGIFKIIRKRLGLILFSALIVTILGGIYTFFIASPVYTASTQLVVKL 58
CapA     ------MKEKFDLVKLLNILKKNIKLLLILPAICLVVSAALTFFVMPDKYTASTQILVNM 54
Wzd GG   ------MNKQIDLSQLWNVFKRSFLAMIVFGIIGMAAAYFGAKAFIAPKYESDTSLLVNR 54
EpsC P   ------MNKQTDLSQLWNVFKRSFLAMVIFGILGLAAAYFGAKTFISPKYESDTSLLVNR 54
EpsC     ------MNKQIDLSQLWNVFKRSFVAMIILGILGMAAAYFGAKTFIAPKYESDTSLLVNR 54
Wzd      ------MNEQIDLARLWNVFKHSFIVMILLGLLGMFIAYFGAKTFIAPKYSASTSMLVNR 54
EpsB     MENSTKTENTIDLRRLWMLLRAHIWSIILWAIGLGAVGFVLAAFVVEPKYTSTTQILVNQ 60
CpsC D39 MKEQNT--IEIDVFQLFKTLWKRKLMILLVALVTGAGAFAYSTFIVKPEYTSTTRIYVVN 58
CpsC     MKEQNT--LEIDVLQLFRALWKRKLVILLVAIITSSVAFAYSTFVIKPEFTSMTRIYVVN 58
Wzd S    MNQDNTKSDEIDVLALLHKLWTKKLLILFTAFYFAVFSFLGTYFFIQPTYTSTTRIYVVN 60
EpsC S   MNQDNTKSDEIDVLALLHKLWTKKLLILFTAFYFAVFSFLGTYFFIQPTYTSTTRIYVVN 60
                    *:  :   :      ::.        .   :  .    : : * : *

EpsA B40 PN-SDNSAAYAGEVTGNIQMANTINQVIVSPVILDKVRSNLN------------------ 99
EpsA     PN-SDNSAAYAGEVTGNIQMANTINQVIVSPVILDKVQSNLN------------------ 99
EpsA KF  PN-SDNSAAYAGQVTGNIQMANTINQVIVSPVILDKVQSNLN------------------ 99
CapA     KK-SSSDLAFQ-NVQSSLQSVNTYTEIIKSPRILDKVSREFDGQ---------------- 96
Wzd GG   KQ-DNDPNMQLNAQQADIQIINTYKDIITRPVVLQAVAEDLTSPQRVMVKKAKPAVYGTR 113
EpsC P   KQ-DNDPNMQLNAQQADIQIINTYKDIITRPVVLQAVAEDLTSPQRVMVKKSKPAVYGTR 113
EpsC     KQ-DNDPNMQLNAQQADIQIINTYKDIITRPVVLEAVADDLTSPQRVMVKKAKKAVYGTR 113
Wzd      KQ-DNNPNMQLNAQQADIQIINTYKDIITRPVILREVADDLTSPRRVKVKKAQKAVYGTR 113
EpsB     KRNAVDAGQAYNAQQADVQVINTYKDIVTSPVILKDASKWIKNP-TEVVKPAKKAKYKTL 120
CpsC D39 RNQGDKSGLTNQDLQAGSYLVKDYREIILSQDALEKVATNLKLD---------------- 102
CpsC     RDQGEKSGLTNQDLQAGSSLVKDYREIILSQDVLEEVVSDLKLD---------------- 102
Wzd S    QATDNKN-LSAQDLQAGTYLANDYKEIITSNDVLSEVIKDEKLN---------------- 103
EpsC S   QATDNKN-LSAQDLQAGTYLANDYKEIIASNDVLSEVIKDEKLN---------------- 103
              .         ..    :   :::     *  .

EpsA B40 ------------------------------LSDDSFQKQVTAANQTNSQVIMLTVKYSNP 129
EpsA     ------------------------------LSDDSFQKQVTAANQTNSQVIMLTVKYSNP 129
EpsA KF  ------------------------------LSDDSFQKQVTAANQTNSQVITLTVKYSNP 129
CapA     ------------------------------YSTAELNSFLKVTNQTNSQIITVSVTTGNK 126
Wzd GG   YNAVTGTRERYVTEEAQPAKYKLKPAKYSNISEEDLTKMVSVSTQQNSQVFTVNVKDTSP 173
EpsC P   YNSVTGVRERYVTEKAQPAEYKLEPAKYSNISEEDLTKMVSVSTQQNSQVFTVNVKDTSP 173
EpsC     YNATTGVREEYVAEKAQPAKYKLRPAKYSNISADDLAKIVSVSTQQNSQVFTVNVRDTDP 173
Wzd      YNAATGVRERYVVKEEQPAKYKLKPAKYANISEDDLDKMISVSNAQNSQVFTVNVRDTDP 173
EpsB     ADGTK-----KLVRPAEPAVIRRAGRGYN-VSAKEMQKAVSVTTQQQSQVFTISAKSNDP 173
CpsC D39 ------------------------------MPAKTLASKVQVTVPTDTRIVSISVKDKQP 132
CpsC     ------------------------------LTPKDLANKIKVTVPVDTRIVSVSVSDRVP 132
Wzd S    ------------------------------LSEAELSKMVSVNIPTDTRLISISVNAKTG 133
EpsC S   ------------------------------LSEAELSKMVSVNIPTDTRLISISVNAKTG 133
                                        .   : . : .    :::. :..

EpsA B40 YIAKKIADETAKIFSSDAAKLLNVTNVNILSKAKAQTTPISPKPKLYLAISVIAGLVLGL 189
EpsA     YIAKKIADETAKIFSSDAAKLLNVTNVNILSKAKAQTTPISPKPKLYLAISVIAGLVLGL 189
EpsA KF  YIAQKIADETAKIFSSDAAKLLNVTNVNILSKAKAQTTPISPKPKLYLAISVIGGLVLGL 189
CapA     SESDKIVNKISKVFAHDMPKIMSVDNVTILSSAHDNAVKVSPIVSVNLVISIIVGIVLAI 186
Wzd GG   VRARDIANEIAKVFEKKIAKIMSISNVSVVSRATANTIPVSPKLKLLSIVGLALGILIAL 233
EpsC P   VRARDIANEIANVFEKKIAKIMSISNVSVVSKATADPTPVSPKLNLAGLVGLLFGILFAF 233
EpsC     LRARDIANDIAKVFEKKIATIMSISNVSVVSKATATSTPVSPRLKLMTIVGLVLGVLVAF 233
Wzd      VRAKDVANEIAKVFKAKIASIMSVSNVSIVSRATADPTPVTPNLKIASLIGLILGMVLAF 233
EpsB     EKSQAIANAVAQTFKNKIKSIMNVNNVTIVSPASVG-AKTFPKTTLFTLAGVVLGLIISV 233
CpsC D39 EEASRIANSLREVAVEKIVAVTRVSDVTTLEEARPATTPSSPNVRRNSLFGFLGGAVVTV 192
CpsC     EEASRIANSLREVAAQKIISITRVSDVTTLEEARPATSPSSPNIKRSTLIGFLAGVIGTS 192
Wzd S    QDAQTLANKVREVASKKIKKVTKVEDVTTLEEAKLPESPSSPNIKLNVLLGAVLGGFLAV 193
EpsC S   QDAQTLANKVREVASKKIKKVTKVEDVTTLEEAKLPESPSSPNIKLNVLLGAVLGGFLAV 193
           :  :.:   :    .   :  : :*. :. *        *        .   * .
```

```
EpsA B40 AIALLKELFDNKINKEEDIEA-LGLTVLGVTSYDQMSDFNKNTNKNGTQSGTKSSPPSDH 248
EpsA     AIALLKELFDNKINKEEDIEA-LGLTVLGVTSYDQMSDFNKNTNKNGTQSGTKSSPPSDH 248
EpsA KF  AIALLQELFDNKINKEEDIEA-LGLTVLGVTTYAQMSDFNKNTNKNGTQMGTKSSPPSDH 248
CapA     LIIFLKELLDKRIKTEEDVESQLGLPILGS-----IQKF--------------------- 220
Wzd GG   SWGLVRELTDQTIKDIDFITDDLGLVNLGIVNYVVRMKDMDQAIQQSRATDSGNDVQDDL 293
EpsC P   AWGLIRDLTDQTIKEIDFITDTLGLVDLGAVNYVRRMKDMDQAIEESKTKLQNNSDSFED 293
EpsC     IWGLIRELTDQTIKDIDFITDDLGLVNLGIVNYVQRMRDMDQAIEAVKSNEDNN----DF 289
Wzd      TVGLIRELTDQTIKSIDFITNDLGLVNLGLVNYVQRMNDMDEAIARSKNKIIDS--EAEP 291
EpsB     ALIILRDSFNTTVRDDDYLTKELGLTNLGHVSHFHLSNKFSINNNDN------------- 279
CpsC D39 IAVLLIELFDTRVKRPEDIEDVLQIPLLGLVPDLDKMK---------------------- 230
CpsC     VIVLILELLDTRVKRPKDIEDTLQMTLLGIVPNLNKLK---------------------- 230
Wzd S    VGVLVREILDDRVRRPEDVEDALGMTLLGIVPDTDKI----------------------- 230
EpsC S   VGVLVREILDDRVRRPEDVEDALGMTLLGIVPDTDKI----------------------- 230
            :: :  :  :.  . :   * :  **

EpsA B40 EVNRSSKRNKR-- 259
EpsA     EVNRSSKRNKR-- 259
EpsA KF  EVNRSSKRNKR-- 259
CapA     -------------
Wzd GG   DGIDFPQRSRRRI 306
EpsC P   EEPDFPRRSRRRV 306
EpsC     GEADFPQRSRRRI 302
Wzd      ETTGFPQRSRRRV 304
EpsB     ---SFGKKKRV-- 287
CpsC D39 -------------
CpsC     -------------
Wzd S    -------------
EpsC S   -------------
```

Figure S2. Alignment of polysaccharide co-polymerases of thePCP2b subclass.

Wzd from *L. rhamnosus* RW-9595M and ATCC 9595 (Wzd) [GenBank: AAW22487.1] were compared with homologous proteins including Wzd from *L. rhamnosus* GG (Wzd GG) [GenBank: CAR87948.1], EpsC from *L. paracasei* (EpsC P) [GenBank: WP_016383925], EpsC from *L. casei* LOCK919 (EpsC) [GenBank: YP_008200731], EpsB from *L. johnsonii* F19785 (EpsB) [GenBank: CAX67043.1], EpsA from *L. lactis* subsp. cremoris JFR1 (EpsA) [GenBank: AER51660.1] or NIZO B40 (EpsA B40) [GenBank: NP_053033.1], EpsA from *L. lactis* subsp. lactis KF147 (EpsA KF) [GenBank: ABX75679.1], CapA from *Staphylococcus aureus* (CapA) [GenBank: AAD52053.1], Wzd from *S. thermophilus* MR-2C (Wzd S) [GenBank: AAL32496.1], EpsC from *S. thermophilus* Sfi6 (EpsC S) [GenBank: AAC44010.1] and CpsC from *S. pneumoniae* D39 (CpsC D39) [GenBank: ABJ55335.1] or from *S. pneumoniae* Rx1-19F (CpsC)

[GenBank: AAC44960.1]. Regions corresponding to the motifs associated with transmembrane sequences are highlighted in yellow and the coiled-coil region is underlined. Tyrosines are in red. Alignments were performed using the program ClustalW2 (http://www.ebi.ac.uk/Tools/msa/clustalw2/) and transmembrane prediction was carried out with TMpred (http://www.ch.embnet.org/software/TMPRED_form.html).

References

1. Péant B, LaPointe G, Gilbert C, Atlan D, Ward P, Roy D: **Comparative analysis of the exopolysaccharide biosynthesis gene clusters from four strains of *Lactobacillus rhamnosus***. *Microbiology* 2005, **151**(6):1839-1851.
2. Aravind L, Koonin EV: **Phosphoesterase domains associated with DNA polymerases of diverse origins**. *Nucleic Acids Res* 1998, **26**(16):3746.
3. LaPointe G, Atlan D, Gilbert C: **Characterization and site-directed mutagenesis of Wzb, an O-phosphatase from *Lactobacillus rhamnosus***. *BMC Biochem* 2008, **9**(1):10-.
4. Hofmann K, Stoffel W: **TMbase-A database of membrane spanning protein segments**. *Biol Chem Hoppe-Seyler* 1993, **374**:166.
5. Brendel V, Bucher P, Nourbakhsh IR, Blaisdell BE, Karlin S: **Methods and algorithms for statistical analysis of protein sequences**. *Proc Natl Acad Sci* 1992, **89**(6):2002-2006.
6. Vincent C, Doublet P, Grangeasse C, Vaganay E, Cozzone AJ, Duclos B: **Cells of *Escherichia coli* contain a protein-tyrosine kinase, Wzc, and a phosphotyrosine-protein phosphatase, Wzb**. *J Bacteriol* 1999, **181**(11):3472.

7. Glucksmann M, Reuber T, Walker G: **Genes needed for the modification, polymerization, export, and processing of succinoglycan by *Rhizobium meliloti*: a model for succinoglycan biosynthesis**. *J Bacteriol* 1993, **175**(21):7045.

8. Tocilj A, Munger C, Proteau A, Morona R, Purins L, Ajamian E, Wagner J, Papadopoulos M, Van Den Bosch L, Rubinstein JL *et al*: **Bacterial polysaccharide co-polymerases share a common framework for control of polymer length**. *Nature Structural and Molecular Biology* 2008, **15**(2):130-138.

9. Morona R, Purins L, Tocilj A, Matte A, Cygler M: **Sequence-structure relationships in polysaccharide co-polymerase (PCP) proteins**. *Trends Biochem Sci* 2009, **34**(2):78-84.

10. Morona R, Van Den Bosch L, Daniels C: **Evaluation of Wzz/MPA1/MPA2 proteins based on the presence of coiled-coil regions**. *Microbiology* 2000, **146**(1):1-4.

11. Doublet P, Vincent C, Grangeasse C, Cozzone A, Duclos B: **On the binding of ATP to the autophosphorylating protein, Ptk, of the bacterium *Acinetobacter johnsonii***. *FEBS Lett* 1999, **445**(1):137-143.

12. Grangeasse C, Doublet P, Cozzone AJ: **Tyrosine phosphorylation of protein kinase Wzc from *Escherichia coli* K12 occurs through a two-step process**. *J Biol Chem* 2002, **277**(9):7127.

13. Morona JK, Paton JC, Miller DC, Morona R: **Tyrosine phosphorylation of CpsD negatively regulates capsular polysaccharide biosynthesis in *Streptococcus pneumoniae***. *Mol Microbiol* 2000, **35**(6):1431-1442.
